# Prospective memory tasks: a more sensitive method for screening cognitive impairment in ALS?

**DOI:** 10.1186/1471-2377-12-142

**Published:** 2012-11-21

**Authors:** Ying Ji, Ling Wei, Dehua Chui, Kai Wang, Dongsheng Fan

**Affiliations:** 1 Department of Neurology, Peking University Third Hospital, Beijing, China; 2 Department of Neurology, Anhui Medical University First Hospital, Hefei, China; 3 Neuroscience Research Institute, Peking University, Beijing, China

## Abstract

****Background**:**

Cognitive change is prevalent in patients with amyotrophic lateral sclerosis (ALS), but still lack a widely accepted and sensitive screening method. In this study, we try to find a sensitive screening battery for detecting subtle cognitive deficits in patients with ALS.

**Methods:**

Eighty consecutive ALS patients and 57 matched normal controls underwent the Mini-Mental Status Examination (MMSE), the verbal fluency test (VFT), the Stroop Color Word Interference Test (CWT), and the prospective memory (PM) tests, including event-based (EBPM) and time-based (TBPM).

**Results:**

The patients did not differ from the controls in the MMSE, the VFT and the CWT. By contrast, statistically significant differences were found in the PM tests (EBPM: P=0.043; TBPM: P<0.001). More interestingly, TBPM was more sensitive than EBPM in the early-phase patients.

**Conclusions:**

Prefrontal lobar dysfunction does exist among ALS patients and may spread from the medial to the lateral region. The PM tests seem more sensitive in ALS patients with frontotemporal dysfunction than are the classical cognitive measures.

## Background

Amyotrophic lateral sclerosis (ALS) is now considered a multisystem disease that co-occurs with frontotemporal dysfunction. A majority of studies on cognitive function have suggested that ALS patients suffer from varying degrees of impairment in different fields of cognitive functioning. The prefrontal cortex (PFC) is the area most involved in early-stage ALS patients with frontotemporal dysfunction [1,2]. There are three principal circuits in frontal lobe for cognitive process, the dorsolateral, ventromedial and orbitofrontal [3]. Among them the dorsolateral-caudate nucleus circuit related to the executive functions has been the most investigated in ALS patients [4,5]. However, no widely accepted and sensitive screening tool is available for early detection because traditional neuropsychological examinations can have normal results in early-stage ALS patients. Memory abnormalities have been less well characterized, and previous studies have mostly focused on retrospective memory (RM), although prospective memory (PM) is now gaining attention. PM is the ability to carry out intended actions in the future, it requires subjects to execute some activities at an appropriate time point, either upon the occurrence of a specified stimulus in the environment (event-based PM, EBPM) or at a special point in time (time-based PM, TBPM), while the subject is engaged in an ongoing, attention demanding activity. It contains two components: a prospective and a retrospective component. The prospective component involves remembering that one needs to do something in the future whereas retrospective component is to remember the content of the action that is supposed to be performed. [6,7] Converging evidence from a number of neuropsychological, electrophysiological and neuroimaging studies has indicated that the PFC is the core area for PM, but varies with different subtypes [8,9]. A meta-analysis of Brodmann area 10 coritco-function also showed that specific part of rostral PFC associated with different cognitive processes (i.e. working memory, episodic memory retrieval, mentalizing ) is all required by PM task [10,11]. For this reason, our study attempted to determine if assessing PM can lead to a sensitive ALS index.

## Methods

### Subjects

Eighty-nine consecutive patients diagnosed with ALS according to the EI Escorial criteria [12] were recruited from the neurology department of Peking University Third Hospital. Exclusion criteria included those with severe depression or anxiety [Hamilton Depression Rating Scale (HAMD) > 20 or Hamilton Anxiety Rating Scale (HAMA) > 8], or with abnormal respiratory function [forced vital capacity (FVC) < 50%], or with upper limb involvement that cannot complete PM tasks. The study ultimately included an 80-patient cohort. The disease severity was evaluated by the ALS Functional Rating Scale (ALSFRS) [13]. Based on the clinical symptoms, 23.8% of the patients were bulbar onset, and 76.3% were spinal onset. The patients in our study were also classified according to various diagnostic levels. The patients diagnosed with clinically definite or clinically probable ALS were classified as advanced-phase ALS (ap-ALS), while clinically probable-laboratory supported and clinically possible ALS were classified as early-phase ALS (ep-ALS) [14]. Fifty-seven age-, sex-, and education-matched healthy adults were also recruited for this study. The ethical committee of Peking University Third Hospital approved the present study. The study design was thoroughly explained and written information was provided to each subject, and if they agreed to participate, an informed consent form according to the Declaration of Helsinki was signed.

### PM task

PM was assessed using a standard Chinese version of the PM test designed by Einstein and McDaniel [7,15]. The test was divided into 2 parts.

1) In the EBPM, 32 pictures (the first 2 pictures were practice items) were presented to the participants. Twelve frequently used Chinese nouns were on each picture. Ten of the nouns belonged to a certain category, and the other 2 belonged to another group (e.g., fruit, city, color). The participants were asked to pick the 2 distinctive nouns and read them. The accuracy rate was recorded. If the 2 nouns were animals (target words), the participants were instructed to perform the EBPM task (patting their legs). There were 7 pairs of target words, and they appeared on the 2nd (for practice), 6th, 11th, 16th, 21st, 25th and 31st slides. One point was scored for each correct selection. After the 32nd slide, the participants were asked to say their telephone numbers immediately (2 points). The maximum total score on the EBPM test was 8. Next, the participants were asked to recall all of the animals they had just picked as a simple RM index called RM1.

2) In the TBPM, the participants were asked to select the largest and the smallest numbers from a series of pictures. Twelve numbers were on each picture. They were instructed to pat their legs every 5 minutes (at 5, 10, and 15 minutes from the beginning of the test). Two points were scored for patting their legs for less than 10 seconds after or before the exact time and 1 point for patting them longer than 10 seconds but less than 30 seconds. A table clock was placed beside the screen. A maximum of 6 points were possible in this part. Also the participants were asked to repeat the three time points, and the results were used as their RM2.

After finishing each part, the participants were asked to recall the instruction as accurately as possible.

### Procedures

The ALS patients and controls completed a series of assessments. The Mini-Mental Status Examination (MMSE) was administered for global assessment of cognitive function. Executive functioning was estimated by a verbal fluency test (VFT) for categories (animals and fruits) and by the Stroop Color Word Interference Test (CWT). In addition, the PM task was completed by the participants.

### Data analysis

The data that followed a normal distribution were characterized by the mean (SD) and compared using an independent T test; otherwise, the median (interquartile range (IQR)) and the Mann–Whitney U test were used. Significance was defined as a *p* value < 0.05. Spearman’s rank-order correlations were also computed for the patients. The statistical analyses were performed using SPSS 15.0 software.

## Results

No significant demographic differences were found between the ALS patients and controls, including age, sex and educational background. Due to dysarthria and mild color-blindness, 74 patients completed the VFT, while 69 patients and 55 controls finished the CWT. Table 1 summarizes the results of executive functioning tests. The ALS patients performed equivalently on MMSE, VFT and CWT compared to the controls. Due to illiteracy, only 74 patients and 52 controls completed the PM task. The results indicate that the ALS patients performed significantly more poorly on both the EBPM (z=−2.027, *p*=0.043) and TBPM (z=−3.782, *p*<0.001) tests (Table 2). Further comparisons showed that the ap-ALS patients (n=27) demonstrated deficits in both EBPM (z=−3.292, *p*=0.001) and TBPM (z=−2.544, *p*=0.011), but the ep-ALS patients (n=47) had TBPM deficits only (z=−3.937, *p*<0.001). We did not find difference on PM task between bulbar onset and spinal onset participants or groups of bulbar involved and not. Meanwhile ALS patients had lower score on RM2 than controls, but all the participants can recall the instructions correctly.

**Table 1:** The demographic data and neuropsychological test results for the ALS patients and controls

	**Patients**	**Controls**	***p***
Age, y	53.1 (12.1)	50.9 (10.0)	0.247
Education, y	12 (9–15)	12 (9–14)	0.118
Male (%)	51 (63.8)	28 (49.1)	0.088
ALSFRS	40.5 (34–43)	-	-
MMSE	29 (27–30)	29 (28–30)	0.139
VF (animal)	16.0 (12.8-19.3)	17.0 (14.0-20.5)	0.192
VF (fruit)	11.0 (9.0-13.3)	11.0 (10.0-14.5)	0.131
CWT	8.0 (5.0-12.5)	10.0 (7.0-15.0)	0.126

**Table 2:** The PM task results for the ALS patients and controls

	**Patients**	**Controls**	***p***
EBPM	6 (4–8)	7 (5.25-8)	0.043
RM1	3 (2–4)	3 (2–4.75)	0.931
Accuracy rate of EBPM (%)	100 (96.7-100)	100 (100–100)	0.364
TBPM	6 (4–6)	6 (6–6)	<0.001
RM2	6 (6–6)	6 (6–6)	0.016
Accuracy rate of TBPM (%)	91.3 (87.5-94.1)	91.3 (89.4-93.9)	0.582

In the correlation analysis, the TBPM did not correlate significantly with the ALSFRS or executive functioning test scores within the patient group, but the association between the EBPM and VFT scores was significant (animals: r = 0.382, *p* = 0.001; fruits: r = 0.417, *p*<0.001).

## Discussion

Frontal lobe dysfunction is the most central part of ALS patients with cognitive change, though area beyond the frontal lobe may also be involved [16]. This point of view has been widely accepted and confirmed by a number of various types of studies. A structural imaging study found most of the frontal regions, including left middle and inferior frontal gyrus and the medial premotor cortex, were significantly more atrophy in ALS/FTD group than pure ALS patients [2]. Another PET study showed that hypometabolism only confined in frontal lobe in FTD/MND compared to FTD [1]. Thus examinations related to frontal lobe are being a relative topic in this area. Consistent with prior studies, the MMSE was not a sensitive screening index for frontotemporal dysfunction [17]. There are some screen batteries focused on executive function appear to be more effective such as Frontal Assessment Battery (FAB) [18], but still lack consistent evidence. Nevertheless, at least one global cognitive screening instrument should be used with ALS patients to rule out other causes of cognitive impairment [19], and the patients’ physical disabilities must also be noted. More detailed neuropsychological assessments should subsequently be performed. Unexpectedly, we found that the Chinese ALS patients performed as well as the controls in the VFT and CWT. Executive function, particularly as assessed by the VFT, is considered to be a sensitive indicator for detecting ALS cognitive impairment in most studies performed in Western countries [20]. Our study implies that, due to cultural differences or other reasons, the VFT might not be appropriate for ALS patients in China. However, the ALS patients had greater deficits in the PM task without traditional executive functioning impairment.

RM impairment has not been consistently observed in ALS patients with cognitive deficits. To the best of our knowledge, this is the first study to examine PM impairment in ALS patients. PM has recently been used in a wide range of research studies and in clinical practice for many kinds of diseases, especially those associated with the PFC, such as schizophrenia and Alzheimer disease, both of which had impaired PM performance [21,22]. The link between rostral PFC and PM was initially noted from brain lesion studies that some researchers found patients who suffered frontal lobe lesion had impaired PM [23]. The first neuroimaging study which carried out in 1998 confirmed the relation between rostral PFC and PM, and then was evidenced by a series of researches [8,24]. Besides, ERP study on normal aging has also suggested that declining PM is the product of diminished frontal function [9]. Combined with behavior researches [25], it seems to be a secure finding that performance of PM is typically accompanied by activations within rostral PFC so far [26]. Therefore, the present study confirms that ALS patients do have PFC impairment. More importantly, it provides evidence for our prediction that compared to traditional neuropsychological examinations, PM tests might serve as a more sensitive screening battery for detecting subtle cognitive changes earlier.

More interestingly, the ap-ALS patients demonstrated deficits on both the EBPM and TBPM, but the ep-ALS patients performed significantly more poorly on the TBPM only. It seems likely that the EBPM and TBPM do not always decline in parallel. This dissociation in PM impairment has been suggested by a number of studies, such as one that applied PM tasks to patients with Parkinson disease and found that the patients had impaired EBPM, but not TBPM [15,27]. These findings suggest that the two PM subtypes are mediated by different neural networks. This phenomenon may be explained by the gateway theory of Burgess *et al*[28]. This hypothesis states that the rostral PFC plays a role in switching between stimulus-independent thought (SIT) and stimulus-oriented thought (SOT). The lateral PFC is crucial for maintaining attention toward internally generated thoughts, including attention state switching between SIT and SOT, while the medial PFC plays a role in attention tasks directed toward external stimuli. The research of Okuda *et al*. suggests that frequent attention switching between performing ongoing activities and focusing on EBPM tasks is more closely related to the lateral area of the rostral PFC. By contrast, ongoing activities and watching the clock to check the time are both exhibited externally in the TBPM task, implying a correlation between the medial PFC cortex and TBPM tasks [11]. Based on this view, our observation that the EBPM test scores showed greater distinction between the ep-ALS and ap-ALS patients than did the TBPM test scores could be interpreted as indicating that the PFC dysfunction in ALS patients may have spread from the medial to the lateral PFC. Moreover, the correlation analysis found that only the EBPM was correlated with the VFT, which relies more on dorsolateral PFC; this result also supports our interpretation.

There was also a larger difference between the two groups in RM2 than in RM1. This result may not indicate that the ALS patients had poor RM compared to the controls because the RM2 task was easier than the RM1 task on which the ALS patients performed comparably to the controls. Meanwhile the instruction recall was fairly well in patients group also revealed the relative preserved retrospective component. It is not clear whether this difference can be attributed to difficulties in time perception, and it requires further study.

Our study did not find difference on PM task between bulbar and spinal onset or bulbar involved and not. The association between cognitive changes and bulbar onset has been suggested by some studies [29,30], but still under controversial [31]. The relative small sample size of bulbar onset group maybe one of the reason of our negative result. Therefore it is cautious to make final conclusion and needs more research to clarification.

This study has certain limitations. First, our ALS patients exhibited equivalent performances on traditional executive examinations, but the finite tests chosen and the patients with severe depression and anxiety or severe dysarthria were excluded may have influenced the results. Second, this study using a simple PM task not based on any imaging examination is very preliminary, but we are inspired by the result. It is a hope that this study may draw attention on this field, and more complex PM task combined with imaging or electrophysiological method could be carried in the future. Third, this study was a cross-sectional study and found that the ALS stage may be a predictor of cognitive functioning. Long-term follow-up studies should be conducted. Therefore, we urge caution when drawing strong conclusions until further research clarifies or verifies the findings in this study.

## Conclusions

In summary, using comparatively systemic neuropsychological tests, our study confirms that Chinese ALS patients have cognitive impairments. PFC dysfunction does exist and may have spread from the medial to the lateral part. The PM tasks seem more sensitive than the traditional neuropsychological measures for detecting cognitive dysfunction in patients with ALS.

## Competing interests

We declare that we have no conflict of interest.

## Authors’ contributions

YJ carried out the neuropsychological measures and drafted the manuscript. LW carried out the neuropsychological measures. DC participated in the design of the study and performed the statistical analysis. KW conceived of the study, and participated in the design of the study. DF conceived of the study, participated in its design and coordination and helped to draft the manuscript. All authors read and approved the final manuscript.

## Pre-publication history

The pre-publication history for this paper can be accessed here:

http://www.biomedcentral.com/1471-2377/12/142/prepub
